# A Theoretical Analysis of the Frequency Response in p-i-n Photodiodes that Use InGaAs/InP Materials

**DOI:** 10.3390/mi16070764

**Published:** 2025-06-29

**Authors:** Nesrine Bakalem, Abdelkader Aissat, Samuel Dupont, Faouzi Saidi, Mohamed Houcine Dhaou, Jean Pierre Vilcot

**Affiliations:** 1 LATSI Laboratory, University Blida1, Blida 09000, Algeria; bakalemnesrine@gmail.com; 2 Department of Material Science, Faculty of Material Science, Mathematics and Computer Science, University of Ahmed Draya, Adrar 01000, Algeria; 3 Institute of Electronics, Microelectronics and Nanotechnology (IEMN), UMR CNRS 8520, University of sciences and Technologies of Lille, 1Avenue Poincare, 60069, 59652 Villeneuve of Ascq, France; samuel.dupont@uphf.fr (S.D.); jean-pierre.vilcot@univ-lille.fr (J.P.V.); 4 Micro-Optoelectronics and Nanostructures Laboratory, Department of Physics, Faculty of Sciences of Monastir, University of Monastir, Environment Street, Monastir 5000, Tunisia; faouzi.saidi@gmail.com; 5 Department of Physics, College of Science, Qassim University, P.O. Box 51452, Burayda Almolaydah 6644, Saudi Arabia; m.dhaou@edu.sa

**Keywords:** materials, semiconductors III-V, p-i-n photodiode, detection, optoelectronic

## Abstract

This investigation is centered on the analysis of frequency response characteristics of a p-i-n photodiode using InxGa_1−x_As/InP. The InGaAs/InP can be developed under three conditions: compression, tensile strain, and lattice matching. Initially, we performed calculations on strain, bandgap energy (E_g_), and absorption coefficient. We then optimized the influence of indium concentration (x) on stability, critical thickness, bandgap energy, and absorption coefficient. The effects of temperature and deformation on E_g_ were also studied. Finally, we optimized the cutoff frequency (f_c_), capacitive effects, and response frequency by considering the impact of x, active layer thickness (d), and surface area (S). For our future endeavors, we intend to explore additional parameters that may affect the p-i-n response. In future work, we can add transparent double layers in the i. InGaAs layer to reduce the transit time, leading to the development of an ultrafast photodiode.

## 1. Introduction

Electrical energy, in essence, transforms an optical signal into an electrical signal. The output voltage varies in proportion to the incoming optical power. A receiver of an optical telecommunication system shall include an optical detector. Such a detector must have high sensitivity to the wavelength of the incident signal, high bandwidth (or high response speed), and linear frequency response (f_r_) to maintain the form of the input signal, low additional noise, and high quantum efficiency. The semiconductor device that aligns with these specifications is the p-i-n photodiode. The characteristics of III-V semiconductors, such as GaAs, InAs, InP, and InGaAs, are highly desirable at a rapid pace in electronics and optoelectronics. The properties of these materials are very interesting for the performance of these devices. In this context, the interaction of light leads to the creation of charge carriers, which, if they do not recombine, can amplify the current of the component. Consequently, pn and p-i-n junctions are extensively utilized as optical devices.

In the depletion layer created between the two regions, P and N, the electric field is responsible for the separation of electron–hole pairs. Furthermore, an increase in the size of the depletion layer corresponds to a greater extent of light absorption. On the other hand, to have a higher electric field, a thin depletion layer is required [1]. In a relatively quiet manner, a thin depletion absorbent region layer is needed to decrease the photogenerated carriers’ transit time. However, a reduced absorptive layer d augments the capacitive effect, which significantly limits the global f_r_ of the photodiode. The influence of these two factors significantly affects the response; the efficiency of the device and its f_r_ can be impacted by this effect.

Various studies have thoroughly examined the f_r_ of p-i-n photodiodes [1,2,3,4,5]. The evaluation of the f_r_ for a typical photodiode has been successfully completed [6]. In the linear scenario, a theoretical investigation was conducted that concentrated on the non-linear response case. The influence of space charge effects on the photodiode under conditions of elevated luminosity was successfully achieved [7,8,9,10]. An analytical model was provided to compute the photodiode f_r_. The f_r_ optimization using two transparent layers implanted on the p-side and n-side of the absorbent area was achieved [1] in order to improve the photodiode response. Authors in [11] have investigated the p-i-n f_r_ composed of a dual depletion region by inserting an intrinsic layer between the active layer and the n+-contact. The temperature can also affect the f_r_ [12]. Finally, ref. [13] developed a mathematical model and compared it with different models in order to show the benefit of using an additional transparent layer instead of using a conventional photodiode. Recent studies show that InGaAs p-i-n photodiodes have become increasingly popular because of their versatile characteristics and extensive applications. These attributes allow for the creation of highly sensitive, ultra-fast, and compact components suitable for various sectors, including high-speed optical telecommunications, medical technology, and advanced electronics and optoelectronics [8,14,15,16,17]. In this work, we explore the f_r_ of a photodiode constructed from InGaAs/InP, analyzing the capacitive effects alongside the global response. The outcome of temperature and indium concentration on the Eg was examined. The effects of strain, absorption surface, and d on the photodiode’s response were thoroughly investigated.

## 2. Materials and Methods

The substrate’s lattice parameter, different from that of the epitaxial layer, imposes its lattice parameter on the latter. This difference causes a deformation of the epitaxial layer, either in tension or in compression by biaxial deformations (ε_zz_) and uniaxial deformations (ε_xx_ = ε_yy_). The present work is based on the In_x_Ga_1−x_As/InP structure, which can exhibit the three cases: lattice match, tensile strain, and compressive strain. These cases are well determined by the mean of the strain, which is expressed as [18]

(1) $$\varepsilon_{xx}(x)=\frac{a_{InP}-a_{InGaAs}(x)}{a_{InGaAs}(x)}$$

(2) $$\varepsilon_{zz}(x)=-2\frac{C_{12}(x)}{C_{11}(x)}\varepsilon_{xx}(x)$$

where a_InP_ and a_InGaAs_(x) are the lattice constants of InP and In_x_Ga_1−x_As, respectively. C_11_ and C_12_ are the elasticity constants. The E_g_ is the measure of the amount of energy necessary for an electron to move forward, shifting from the valence band (VB) to the conduction band (CB). The E_g_ of In_x_Ga_1−x_As alloy, depending on its composition, is given as [19]

E_g_(x) = x∙E_gInAs_ + (1 − x)∙E_gGaAs_ − b∙x + bx^2^ (3)

where E_g InA_s and E_g GaAs_ are the bandgap energies of InAs and GaAs, and b is the bowing factor; it is 0.43 eV. The variation in E_g_ as a function of temperature can be effectively explained by the Varshni model [20]:

(4) $$E_{g}\left(x,T\right)=E_{g}\left(x,0\right)-\frac{\alpha_{1}.T^{2}}{\beta_{1}+T}$$

α_1_ is an empirical constant, and β_1_ is a constant associated with the Debye temperature and the E_g_ of the material at 0 K. In the situation where strain is absent, the heavy hole (hh) bands and light hole (lh) bands exhibit isotropy and degeneracy at the center of the Brillouin zone, and the spin-split hole band is located at an energy ∆_0_ below these two bands, the barycenter of the VB; consequently, it is ∆_0_ below the top of the VB at k = 0.

(5) $$E_{v,moy}=\frac{E_{hh}+E_{lh}+{∆}_{0}}{3}$$

where E_hh_ and E_lh_ are the energies of heavy holes and light holes, respectively.

The strain effect on both the VB and the CB can be divided into two components. The first is a hydrostatic component; it decreases the E_g_. The second is a shear component; it has the effect of lifting the degeneration of heavy holes and light holes from the top of the VB. In the case of a layer under a biaxial compressive strain, the hydrostatic component grows the average E_g_ between the CB and VB, while the shear component produces the strongly anisotropic VB: the higher energy band hh becomes “heavy” according to k_⊥_ and “light” according to k_//_, while the lower energy band lh becomes “light” according to k_⊥_ and “heavy” according to k_//_ [18]. The energy shifts in the centers of gravity for the VB and for the CB in k = 0 vary proportionally to the strain [18,21]:

(6) $${∆E}_{c}^{Hyd}(x)=a_{c}\left(2\varepsilon_{xx}(x)+\varepsilon_{zz}(x)\right)$$

(7) $${∆E}_{v,av}^{Hyd}(x)=a_{v}\left(2\varepsilon_{xx}(x)+\varepsilon_{zz}(x)\right)$$

where

a_c_ is the hydrostatic strain potential associated with the CB.
a_v_ is the hydrostatic strain potential associated with the VB.

(8) $${∆E}_{hh}^{She}(x)=A{∆}_{0}-B{\delta E}^{She}(x)$$

(9) $${∆E}_{lh}^{She}(x)=C{∆}_{0}+D{\delta E}^{She}(x)+0.5\sqrt{\left[{∆}_{0}^{2}+{∆}_{0}{\delta E}^{She}(x)+E{\left({\delta E}^{She}(x)\right)}^{2}\right]}$$

(10) $${\delta E}^{She}(x)=2b(\varepsilon_{zz}-\varepsilon_{xx})$$

where A = 0.33, B = 0.5, C = −0.16, D = 0.25, and E = 2.25.

b signifies the tetragonal deformation potential.
Using E_v,av_ as the energy reference.

(11) $$E_{v}(x)=E_{v,av}+\frac{{∆}_{0}}{3}+{∆E}_{v,\;av}^{Hyd}(x)$$

(12) $$E_{c}(x)=E_{v,av}+\frac{{∆}_{0}}{3}+{∆E}_{c}^{Hyd}+E_{g}(x)$$

(13) $$E_{g}\left(x\right)=E_{c}(x)-E_{v}(x)$$

(14) $${E_{g}}_{hh,lh}(x)=E_{g}(x)+{\Delta E}_{c}^{hyd}(x)-{∆E}_{v,moy}^{Hyd}(x)-{\Delta E}_{hh,lh}^{She}(x)$$

where

${∆E}_{c}^{Hyd}$ is the CB shift due to the hydrostatic component.
${∆E}_{hh}^{She}$ is the VB shift due to the shear component.
E_ghh_ is the bandgap of hh.
E_glh_ is the bandgap of lh.

To study the effect of dislocation on the structure of the component, it is necessary to simulate this physical phenomenon, which influences stability. We used the following model [22]:

(15) $$h_{c}=\frac{a_{InGaN}}{\beta \times \sqrt{2}\times \pi \times ∆}\times \frac{1-0.25\times \gamma }{1+\gamma }\times Ln\left(\frac{h_{c}\sqrt{2}}{a_{InGaN}}+1\right)$$

∆ is the parametric mismatch.
γ is Poisson’s coefficient.
β is a coefficient that equals 4 for a single layer, 2 for a quantum well, and 1 for super networks.

The absorbent layer is considered transparent to photon energy when it is less than the material’s E_g_ (the photon is not absorbed), and the electrons’ transition to the CB is not probable. The α associated with a photon energy E superior to or equal to E_g_ is given by [23]:

(16) $$\alpha \left(E,x\right)=\alpha_{0}\frac{\sqrt{E-E_{g}(x,T)}}{E}$$

E is the energy of the incident photon, E_g_(x,T) is the E_g_ of the semiconductor, α_0_ is a constant depending on the semiconductor. In high-speed optical communications systems, the photodiode is the main part that allows us to detect incoming information. However, the rate of transmission is the major factor in such systems; high or low rates of transmission are quite associated with the f_r_, which has pushed us to optimize the photodiode f_r_ and simulate the global response as well as its limitations. Indeed, photodiode response is largely limited by two elements of topmost significance: the transit duration and the capacitive impact [1,5]. The first one depends on the depletion area width and the carriers’ velocities as τ_p,n_ = d/ν_p,n_. By considering that carriers are moving at their saturation velocities ν_sat,p,n_, the transit time will be directly dependent on the depletion region d. The second factor depends on the depletion zone width and the junction area as C(x) = S/d·ε_o_ε_r_(x); (ε_o_ is the free space permittivity, and ε_r_(x) is the In_x_Ga_1-x_As relative permittivity (Table 1), depending on the indium concentration (x).

The primary objective of this paper is to examine and analyze the limitations associated with the response of photodiodes, such as the x, the transit time, and the capacitive impact. The indium concentration influences the E_g_, so it influences the α. The transit time and capacitive effect are mainly managed by the space charge region (w), i.e., a small transition duration is obtained by using a thin depletion region; however, a reduced capacitive effect can be achieved in the case of a substantial depletion area. Therefore, there exists a compromise between the transit phenomenon and the capacitive one. The impact of the first one appears in the global response through the transit response component. This component is a function of time, and by using the Fourier transform, it is possible to obtain the f_r_. Nonetheless, the second factor affects the overall response through the capacitive effect.The formula presented below indicates the frequency of coupling at −3 dB as a direct function of both transit time and circuit response time.

(17) $$f_{c}\left(-3\;dB\right)={\left(\tau_{tr}^{2}+\tau_{Rc}^{2}\right)}^{-\frac{1}{2}}$$

where τ_tr_ is the transit time and τ_RC_ is the circuit response time.

It will be compelling to explore a compromise that reduces the τ_tr_ for carriers and diminishes the component’s capacity, enabling high coupling frequencies and operation in the hyper-frequency spectrum.

## 3. The Frequency Response Calculation

**Figure 1:**
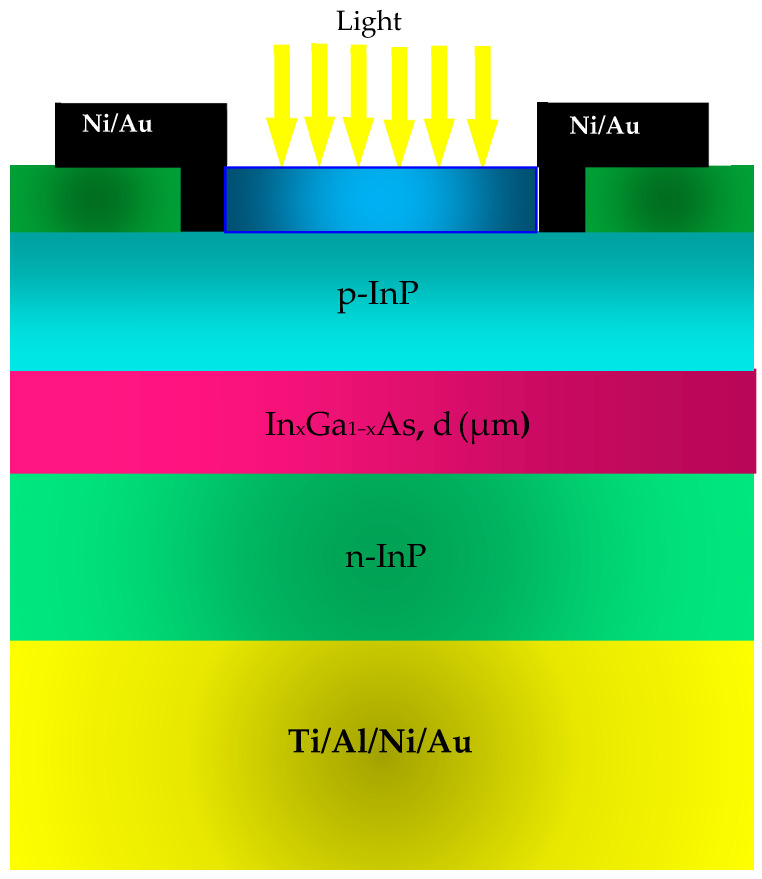
A visual representation of the configuration of the p-i-n photodiode that is being examined.

Figure 1 depicts the configuration of the p-i-n photodiode under investigation; it is composed of an absorbent layer made of InGaAs having a thickness d, placed between an N-doped layer made of InP and a P-doped layer also made of InP. As mentioned previously, this study settled on the assumption that the carriers are traveling with the saturation velocity given at room temperature [21,22,23,24,25,26,27,28,29].

By means of Vegard’s law, it is possible to calculate the In_x_Ga_1−x_As saturation velocity. The overall current in the photodiode is expressed as

(18) $$i\left(t\right)=\frac{q}{d}\left[v_{n}N\left(t\right)+v_{p}P\left(t\right)\right]$$

In this context, d denotes the thickness of the region that is absorbent, q is the electron charge, P(t) signifies the number of holes existing in the absorbent zone, N(t) represents the quantity of electrons present in the absorbent region, and v_p_ and v_n_ represent, respectively, the holes and the electron saturation velocities. The transit responses for both electrons and holes in the time domain represent their participation in the total current and are given by [1,13]. By means of the Fourier transform, the f_r_ associated with the transit component can be obtained. It is considered an essential parameter that describes the performance of an optical communication system; the model is given by [30].

## 4. Results and Discussions

**Figure 2:**
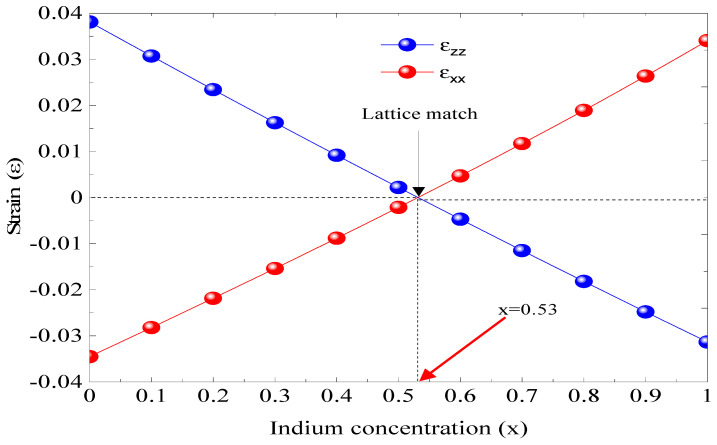
The influence of indium concentration on strain (ε).

In Figure 2, the concentration x impact on the parallel stress (ε_xx_) and the perpendicular stress (ε_zz_) is depicted. The InGaAs/InP structure contains two strains, one compressive when x is less than 0.53 and the second extensive if x is greater than 0.53. However, InGaAs is the lattice matched to InP, i.e., ε = 0, for an indium concentration of 0.53, which represents the intersection between ε_xx_ and ε_zz_; this point represents the robustness of the design (Equations (1) and (2)).

**Figure 3:**
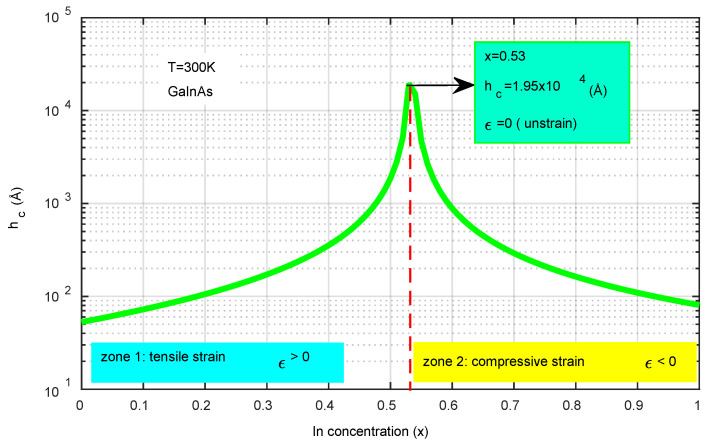
The effect of indium concentration on critical thickness is significant. At x = 0.53, the stress is neutral, indicating structural stability. In zone 1, where x < 0.53, the strain is tensile, meaning ε > 0. Conversely, in zone 2, where x > 0.53, the strain becomes compressive, resulting in ε < 0.

Figure 3 illustrates how the critical thickness (h_c_) varies with the indium concentration (x). The analysis reveals two distinct regions: zone 1, where x is less than 0.53 and the strain is tensile (ε > 0), and zone 2, where x exceeds 0.53 and the strain is compressive (ε < 0). In both regions, the structure exhibits marginal instability, leading to the formation of dislocations. At x = 0.53, the structure achieves maximum stability with no strain (ε = 0). This research provides insights into the optimal indium concentration necessary to prevent structural dislocation. Consequently, the most stable configuration, characterized by no dislocation, is found at x = 0.53 with h_c_ measuring 1.95 × 10^4^ Å. These simulation findings facilitate the optimization of the dimensions for photodiode layers (Equation (15)).

**Figure 4:**
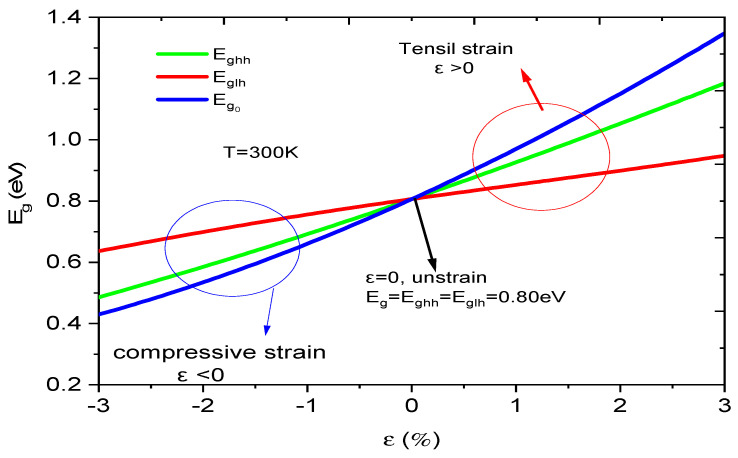
Strain effect on the bandgap energy at room temperature.

Figure 4 exhibits the influence of strain on the E_g_ at room temperature. For unstrained phenomena (ε = 0) with x = 53%, the b and gap energy E_g0_ = E_ghh_ = E_glh_, i.e., there is no valence band splitting. When the strain is matched, the valence band splitting starts to increase, and the E_g_ splits into three: E_g0_, E_ghh_, and E_glh_, respectively (Equations (3) and (14)). This study shows us the impact on the b and bandgap energy and allows us to optimize E_g_ to absorb the maximum of incident photons on the contact surface of the photodiode.

Also, we can control the stability of the component structure. When ε > 0, the strain is extensive, i.e., a_InP_ > a_InGaAs_. On the other hand, if ε < 0, the strain is compressive, i.e., a_InP_ < a_InGaAs_. In order to realize a reliable and stable photodiode, finding a suitable compromise between the E_g_ and the strain is crucial.

**Figure 5:**
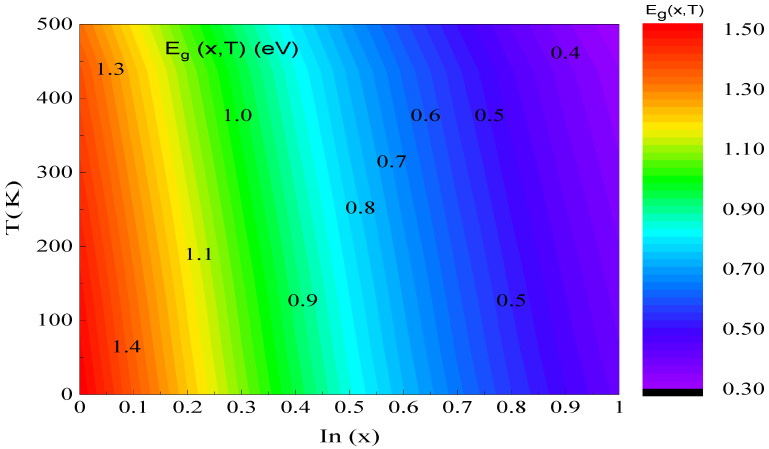
Bandgap energy versus the In concentration and the temperature.

Figure 5 characterizes the evolution of the E_g_ depending on the x using the Varshni model [14]. We have observed that raising the x and temperature results in a significant decline in E_g_. When T = 300 K and x varies from 0 to 1, the E_g_ changes from 1.41 to 0.36 eV, i.e., it decreases by 1.05 eV. We observe that the x significantly influences the development of the E_g_. On the other hand, if we fix the x at 0.53 (unstrained case) and we vary the temperature from 200 to 350 K, the E_g_ fluctuates from 0.76 to 0.72 eV, i.e., the effect of temperature is small compared to the consequence of the x (Equation (4)). This simulation allows us to seek out an agreement between the temperature and concentration impact to develop the efficiency of the proposed photodiode configuration.

**Figure 6:**
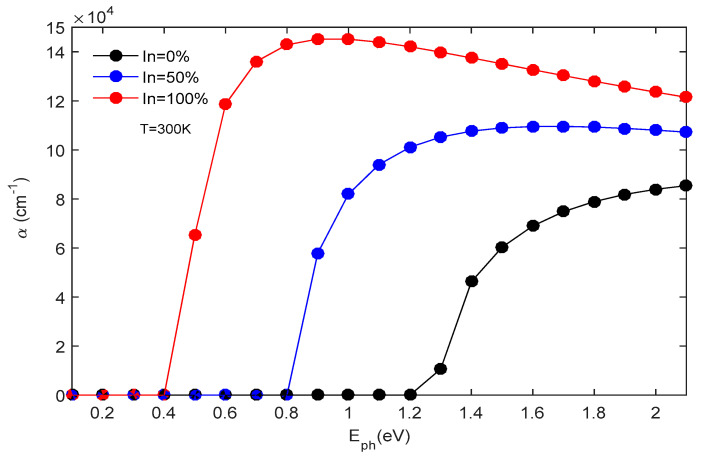
The absorption coefficient’s variation with photon energy is analyzed for various x at a temperature of 300 K.

The impact of incident photon energy and indium concentration x on the α(E,x) at a temperature of 300 K is shown in Figure 6. The proposed structure exhibits an absorption range that spans from 0.40 to 2 eV. When we vary the x from 0 to 1, the amplitude of the α changes from 14.52 × 10^4^ to 8.39 × 10^4^ cm^−1^. Through this simulation, we can determine the most suitable α for our photodiode. The speed of a p-i-n photodiode is primarily influenced by its cutoff frequency (f_c_) at −3 dB. A small capacitive effect is produced for a thick absorber region, which increases the τt and, therefore, degrades the photodiode response; the inverse is true for a thin absorbent region. Thus, there exists a tradeoff between the τ_tr_ and the capacitive effect. Thus, in order to obtain high-speed photodetectors (f_c_ around 60 GHz), it is essential to reduce the τ_tr_ [31,32] and the capacitive consequence. This phenomenon is well illustrated in the section below.

**Figure 7:**
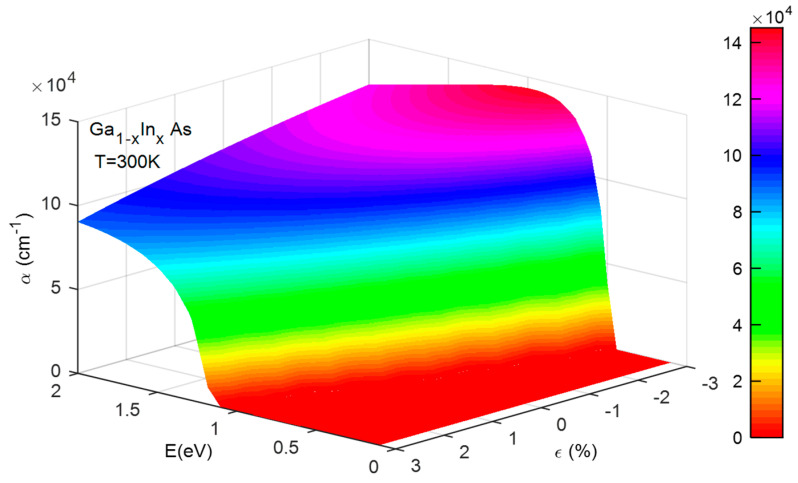
The influence of photon energy and strain on the absorption coefficient of the GaInAs structure at temperature of 300 K.

Figure 7 illustrates how the energy of incident photons and strain (ε) affect the α of the GaInAs structure at room temperature (Equation (16)). The absorption spectrum is observed to range from 0.4 to 2 eV. Additionally, variations in strain significantly impact the α of the component’s active structure. For instance, at a strain level of +3%, indicative of high strain, the optimal α is around 9.14 × 10^4^ cm^−1^. Conversely, at a strain level of −3%, representing compressive strain, the optimal α increases to about 1.40 × 10^5^ cm^−1^. This simulation aids in identifying the α that aligns with the most stable and efficient structure. Our findings indicate that the most stable configuration occurs at ε = 0, corresponding to an α equal to 10^5^ cm^−1^.

**Figure 8:**
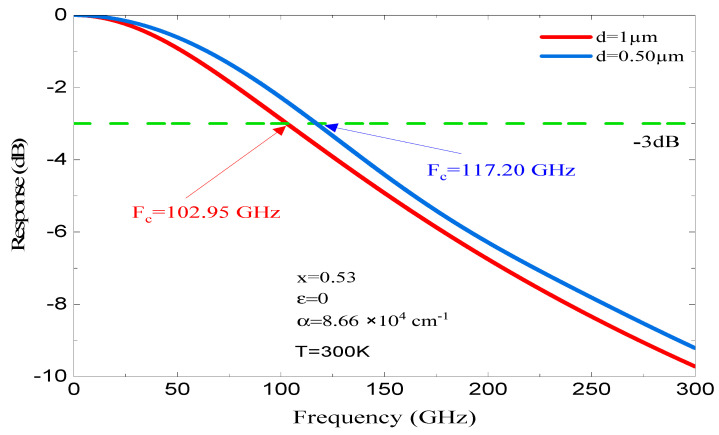
The dependence of f_c_ on d for a lattice-matched structure, characterized by an α of 9.87 × 10^4^ cm^−1^ at room temperature.

**Figure 9:**
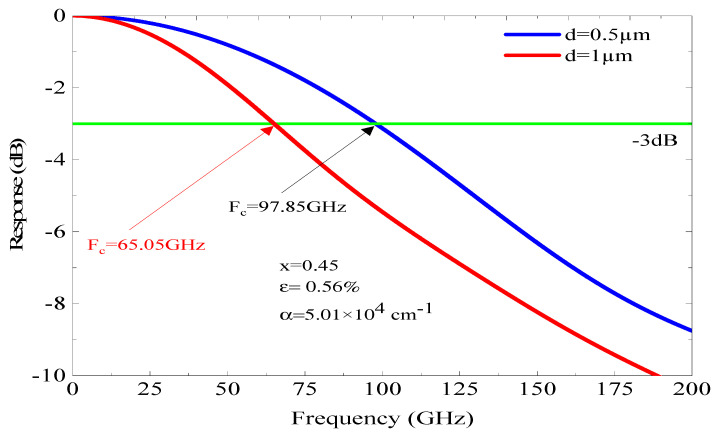
The variation in f_c_ with respect to d, under a tensile strain and α equal 5.0 × 10^4^ cm^−1^ with x = 0.45 and strain ε = 0.56%.

Figure 8 and Figure 9 illustrate how the fc changes with the thickness of the absorber region for x of 0.53 and 0.45, respectively. As shown in Figure 8, for x = 0.53, which corresponds to a lattice-matched structure (stable structure) and an α = 8.66 × 10^4^ cm^−1^. Using an absorbent region thickness d = 1 µm, the fc at −3 dB is 102.95 GHz; however, for the same parameters but different absorbent region thickness (d = 0.50 µm), the fc at −3 dB improves to reach 117.2 GHz. This improvement is due to the absorbent region thickness, which reduces the τ_tr_ effect but gives rise to the capacitive effect. We note that when we reduce the d thickness from 1 to 0.50 µm, the photodiode’s coupling frequency f_c_ (−3 dB) shifts from 102.95 to 117.20 GHz. We found that the frequency of fc increased by Δf_c_ 14.25 GHz. 

This investigation provides the opportunity to refine the thickness d, the x, and the f_c_, thereby enhancing the performance of a photodiode capable of operating at gigahertz frequencies.

**Figure 10:**
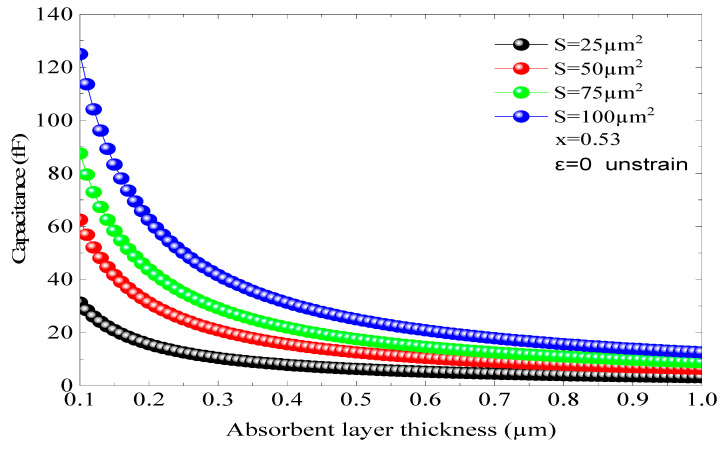
The capacitance changes based on the junction area (S) and distance (d) for x = 0.53 when there is no strain (ε = 0).

Figure 9 depicts the f_c_ of the adopted p-i-n photodiode based on an InGaAs/InP structure with an absorber region thickness of 0.50 and 1 µm and In concentration (x = 45%) that corresponds to a tensile strain of 0.56% and an α of 5.01 × 10^4^ cm^−1^. In this structure, the f_c_ at −3 dB is not important compared to the previous results, despite using the same absorbent region thickness (d). For d = 1 µm, the fc is 65.05 GHz, while it reaches 97.85 GHz for d = 0.50 µm, whereas the improvement in the fc at −3 dB is much more significant than those shown in Figure 8 and Figure 9; it reached 8 GHz. The impact of the absorbent region thickness on the f_r_ can be described by the carriers’ τ and the capacitive effect; however, this latter depends also on the junction area. The variation in the capacitive effect as a function of the junction area (S) and d is illustrated in Figure 10 for a stable structure (x = 0.53 and ε = 0). As n, a low capacitive effect is obtained for low areas and thin absorbent regions. For a surface of µm^2^ and d = 0.50 µm, the capacitance C is equal to 6.25 Fs. Thus, it can be stated that the capacitive effect is limited and does not have an effect on the photodiode performance.

**Figure 11:**
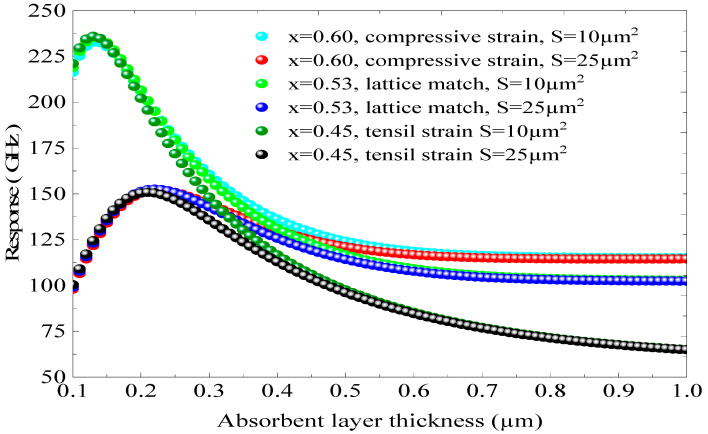
The global frequency response as a function of d and different junction areas (S = 10, 25 µm^2^) using different x.

Figure 11 shows the global frequency response as a function of d and different junction areas, S = 10, 25 µm^2^, using different x, so different absorption coefficients. Figure 11 illustrates the overall frequency response as a function of d and various junction areas, specifically S = 10 and 25 µm^2^, for different values of x: 0.45, 0.53, and 0.60. These values correspond to three distinct conditions: extensively constrained, unconstrained, and compressively constrained, leading to different values of a. When x is set to 0.60 and S is 10 µm^2^, the overall frequency response peaks at 237.5 GHz at d = 0.150 µm. In contrast, for x = 0.60 and S = 25 µm^2^, the frequency response does not surpass 155 GHz. This indicates that an increase in the junction area S significantly reduces the frequency response. A similar trend is observed for the concentrations of 0.53 and 0.45. This simulation provides insights for optimizing S, d, ep, and f_r_ to develop a high-performance p.i.n photodiode functioning within the GHz range. Additionally, we note that the impact of the constraint on f_r_ is minimal.

**Figure 12:**
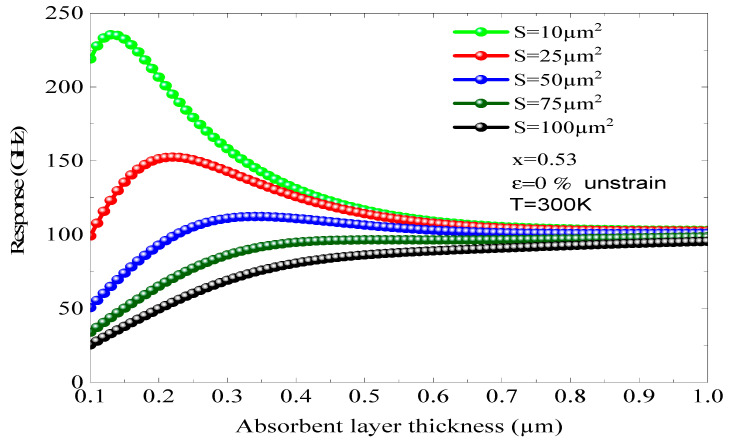
The influence of the thickness of the absorbing layers on the response of the photodiode was examined for different surfaces, with x = 0.53, ε = 0%, and T = 300 K.

The x begins to appear when using a relatively big area. On the other hand, utilizing a small junction zone along with a thin absorbent layer is vital for the decrease in capacity. The variation in the photodiode response based on the thickness of the absorbing layer is illustrated in Figure 12, taking into account different contact surfaces between the incident photons and the photodiode’s face for x = 0.53 and ε = 0, indicating a mesh-matched and stable design. We note that the increase in the contact surface induces a significant decrease in the maximum response of the component. For a surface S = 10 cm^2^, the maximum response of the photodiode reaches 235.34 GHz, with an active layer thickness around 0.15 µm. When the surface is equal to 50 cm^2^, the maximum response of the component decreases rapidly until reaching the value of 112 GHz. Then we note that a degradation of the maximum response of 123 GHz has been reported with an active layer thickness of 0.30 µm, i.e., a decrease of more than 50%. Another phenomenon appearing is the maximum response shifts towards large thicknesses. This analysis provides the opportunity to refine the structure of a compact and ultra-fast optical sensor operating at a response frequency higher than 225 GHz. We find that the maximum response decreases sharply when d is greater than 0.2 µm. To facilitate the operation of a photodiode at frequencies above 200 GHz, an absorbing layer of approximately 0.15 µm must be employed. In addition, it is vital to take into account the phenomenon of structural stability, which directs us to opt for an x equal to 0.53 and a surface area of S = 10 at 25 µm^2^.

## 5. Conclusions

The present research focuses on the f_r_ characteristics of an InGaAs/InP p-i-n photodiode. A simulation has been conducted to assess the impact of the absorbent region’s thickness on the f_c_ (−3 dB), with the most favorable f_r_ identified at a thickness of 0.50 µm. In addition to the influence of the absorbent region thickness, it was also discovered that the f_r_ can be regulated by other parameters, including x, which affects the absorption coefficient. Also, the global response of the photodiode was explored, indicating the effects of the d of the absorber region, the junction area, and the x. The results demonstrate that the best structural configuration occurs at x = 0.53, strain ε = 0%, and E_g_ = 0.80 eV. The optimal design has a frequency response that is about 220 GHz, with a measurement of d = 0.15 µm.

## Figures and Tables

**Table 1:** Some parameters of the binaries that compose the InGaAs and the substrate InP.

Parameters	GaAs	InAs	InP	Ref.
a (Å)	5.65	6.06	5.86	[18]
C_11_ (10^11^ dyn/cm^2^)	11.88	8.33	10.22	[18]
C_12_ (10^11^ dyn/cm^2^	5.38	4.55	5.76	[18]
E_g_, T = 300 K (eV)	1.42	0.35	1.34	[20]
E_g_, T = 0 K (eV)	1.52	0.42	1.42	[18]
α (meV/K)	0.54	0.28	0.36	[21]
β (meV/K)	204	93	162	[21]
$E_{v}\;(eV)$	−6.92	−6.67	−7	[18]
$a_{v}\;(eV)$	1.00	1.16	1.27	[16]
$\Delta_{0}\;(eV)$	0.34	0.43	0.11	[21]
b (eV)	−1.70	−1.80	−2.0	[18]
$a_{c}\;(eV)$	−7.17	−5.08	−5.04	[24]

## Data Availability

The original contributions presented in the study are included in the article, further inquiries can be directed to the corresponding author.
